# Identification of Differential Gene Expression in *Brassica rapa* Nectaries through Expressed Sequence Tag Analysis

**DOI:** 10.1371/journal.pone.0008782

**Published:** 2010-01-20

**Authors:** Marshall Hampton, Wayne W. Xu, Brian W. Kram, Emily M. Chambers, Jerad S. Ehrnriter, Jonathan H. Gralewski, Teresa Joyal, Clay J. Carter

**Affiliations:** 1 Department of Mathematics and Statistics, University of Minnesota Duluth, Duluth, Minnesota, United States of America; 2 Minnesota Supercomputing Institute, University of Minnesota, Minneapolis, Minnesota, United States of America; 3 Department of Biology, University of Minnesota Duluth, Duluth, Minnesota, United States of America; University of Oxford, United Kingdom

## Abstract

**Background:**

Nectaries are the floral organs responsible for the synthesis and secretion of nectar. Despite their central roles in pollination biology, very little is understood about the molecular mechanisms underlying nectar production. This project was undertaken to identify genes potentially involved in mediating nectary form and function in *Brassica rapa*.

**Methodology and Principal Findings:**

Four cDNA libraries were created using RNA isolated from the median and lateral nectaries of *B. rapa* flowers, with one normalized and one non-normalized library being generated from each tissue. Approximately 3,000 clones from each library were randomly sequenced from the 5′ end to generate a total of 11,101 high quality expressed sequence tags (ESTs). Sequence assembly of all ESTs together allowed the identification of 1,453 contigs and 4,403 singleton sequences, with the Basic Localized Alignment Search Tool (BLAST) being used to identify 4,138 presumptive orthologs to *Arabidopsis thaliana* genes. Several genes differentially expressed between median and lateral nectaries were initially identified based upon the number of BLAST hits represented by independent ESTs, and later confirmed via reverse transcription polymerase chain reaction (RT PCR). RT PCR was also used to verify the expression patterns of eight putative orthologs to known Arabidopsis nectary-enriched genes.

**Conclusions/Significance:**

This work provided a snapshot of gene expression in actively secreting *B. rapa* nectaries, and also allowed the identification of differential gene expression between median and lateral nectaries. Moreover, 207 orthologs to known nectary-enriched genes from Arabidopsis were identified through this analysis. The results suggest that genes involved in nectar production are conserved amongst the Brassicaceae, and also supply clones and sequence information that can be used to probe nectary function in *B. rapa*.

## Introduction

Floral nectar is the primary reward offered by angiosperms to attract pollinators [1]. While sugars are generally the dominant solutes, various nectars also contain a multitude of additional chemical components (reviewed in [2]), with some thought to provide supplementary nutrition to pollinators (e.g., [3]), and others appearing to deter visitation by animals with body plans not properly suited for pollen dispersal (e.g., [4]).

**Figure 1:**
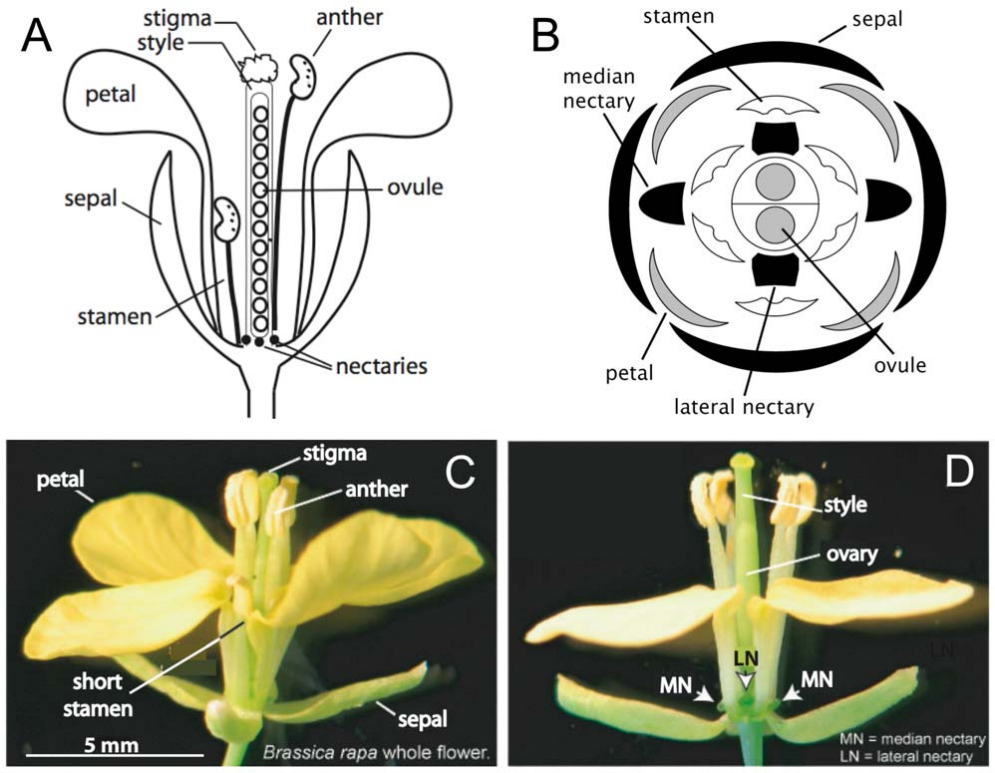
*Brassica rapa* flower structure. (A) Schematic longitudinal section of *B. rapa* flower. (B) Schematic cross-section of flower with relative location of floral organs indicated (modified from [8]). (C) Whole *B. rapa* flower. (D) *B. rapa* flower with one sepal, one short stamen and two petals removed to reveal one lateral (LN) and two median nectaries (MN); lateral nectaries secrete >95% of total nectar carbohydrate, whereas MN produce very little nectar. cDNA libraries made from LN and MN of open, secretory flowers were used for the EST analyses.

The floral organ responsible for generating the complex mix of components in nectar is the nectary. Nectaries are anatomically diverse amongst different species, and have even been used for taxonomic purposes [5]. The flowers of most *Brassica* sp. (e.g., canola, broccoli, cauliflower) contain four nectaries, which consist of two nonequivalent sets of organs known as lateral and median nectaries, respectively [6], [7], [8]. The two lateral nectaries are longitudinally opposed to one another and surrounded by insertion points of long stamens, petals and short stamens (i.e., occur interior to the short stamen, at the base of the filament; Fig. 1) [7]. Median nectaries also occur on opposite sides of the flower but only in between the insertion points of two long stamen [7]. Both lateral and median nectaries are well-differentiated organs and are subtended by phloem, which is thought to provide most “pre-nectar” components (e.g., sucrose, amino acids, etc.) to the nectary [5], [9]. As previously mentioned, median and lateral nectaries are not equivalent – indeed, it is generally thought that only lateral nectaries significantly contribute to the production of nectar in most Brassicaceae species [8], [10], [11], [12], [13].

While the importance of nectaries and nectar in pollination biology is somewhat understood, the molecular mechanisms underlying nectar production are not. To date, only three genes—*CRABS CLAW*, *BLADE-ON-PETIOLE (BOP) 1* and *BOP2*—are known to be required for nectary development [14], [15], and only a single gene, *CELL WALL INVERTASE 4*, has a demonstrated role in *de novo* nectar production [13], [16]. To address this gap in knowledge, we previously performed transcriptomic analyses on RNA isolated from *Arabidopsis thaliana* nectaries [17]. From this study we were able to identify a large number of genes preferentially expressed in nectaries, as well as differential expression patterns between nectary type (lateral vs. median) and developmental stage (pre- vs. post-nectar secretion). This study has allowed reverse genetics to be used to examine nectary function [13], [16]; however, Arabidopsis nectaries are very small (∼100 microns across and deep) and produce very little nectar [8], [13]. Fortuitously, *Brassica rapa* is closely related to Arabidopsis, and produces relatively large amounts of nectar from its much larger nectaries. The study reported here was undertaken in order to identify genes differentially expressed between median and lateral nectaries, as well as putative orthologs of Arabidopsis nectary-enriched genes thought to play roles in nectary form and function.

## Results and Discussion

Nectaries are responsible for the synthesis and secretion of nectar. In order to identify genes potentially involved in *B. rapa* floral nectar production, both normalized and non-normalized cDNA libraries were created using RNA isolated from both median and lateral nectaries (i.e., a total of four independent cDNA libraries were created, see Table 1). When performing expressed sequence tag (EST) analysis, normalized libraries are generally useful for identifying all genes that may be expressed in a given tissue, including ones expressed at low levels [18], whereas non-normalized libraries are better suited for providing an indication of gene expression level by the total number of redundant or overlapping ESTs (i.e., digital expression profiling [19]).

**Table 1:** Summary of *B. rapa* nectary cDNA libraries and resultant ESTs generated for this study.

Library	Tissue source and library type^a^	No. of ESTs^b^	No. of contigs	No. of singletons	Unique Arabidopsis hits^c^
MLN-1	Mature lateral nectary, non-normalized	2,808	337	1,376	1,343
MMN-1	Mature median nectary, non-normalized	2,826	259	1,551	1,253
MLN-2	Mature lateral nectary, normalized	2,582	393	1,206	1,404
MMN-2	Mature median nectary, normalized	2,885	343	1,787	1,860
	All MLN (MLN-1+MLN-2)	5,390	743	2,237	2,342
	All MMN (MMN-1+MMN-2)	5,711	659	2,956	2,704
	All sequences together (unigene set)	11,101	1,453	4,403	4,138

a All nectaries were manually dissected from *B. rapa* flowers at the equivalent of Arabidopsis stage 14–15 flowers (i.e., post-anthesis, nectaries were secretory). Lateral nectaries secrete >95% of total nectar carbohydrate in *B. rapa*, whereas median nectaries produce very little nectar.

b High quality reads >100 bp on inserts.

c Based upon translated searches (blastx) of contigs and singleton sequences against TAIR9 protein annotations.

For library construction, all nectaries were manually dissected from *B. rapa* flowers (an example dissection is available in Video S1), and processed as described in *Materials and Methods*. Each resultant library was examined for quality and had the following characteristics: >1×10^6^ independent clones, average insert size >1,000 bp, minimal cDNA length >500 bp, and >95% recombinant plasmids.

### DNA Sequence Processing and Analysis

Following library quality analysis, approximately 3,000 clones from each cDNA library were randomly sequenced from the 5′ end (single pass sequencing). Each resultant sequence was subsequently trimmed of contaminating vector and linker sequences, generating a total of 11,101 high-quality ESTs (i.e., >100 bp reads on inserts; see Tables S1, S2, S3, S4, S5, S6 & S7). All trimmed sequences were then assembled for each library independently, as well as cumulatively, with a total of 1,453 contigs and 4,403 singleton sequences being identified when all ESTs were analyzed together, resulting in a unigene set of 5,856 total unique, nonoverlapping sequences (see Table 1 and Table S7). Contig and singleton sequences were then subjected to Basic Local Alignment Search Tool (BLAST) [20] analysis via translated searches (blastx) for each library independently, as well as cumulatively, against the most recent Arabidopsis genome protein annotation (TAIR9 proteins, released June 20, 2009 [21]). Results from the BLAST analyses are summarized in Table 1, with full details available in Tables S1, S2, S3, S4, S5, S6 & S7. BLAST analyses on the unigene set identified putative orthologs to 4,138 distinct Arabidopsis genes, with 315 out of 5,856 total input sequences (∼5.7%) not producing significant hits.

### Arabidopsis Nectary-Expressed Genes Represented by *B. rapa* ESTs

Of the 4,138 Arabidopsis orthologs represented by *B. rapa* EST hits, 3,678 (89%) were previously found to be expressed in Arabidopsis nectaries via microarray profiling [17]. The 460 Arabidopsis genes represented by *B. rapa* EST hits, but not confidently expressed in Arabidopsis nectaries, are highlighted in Table S8. Gene ontology (GO) analysis of these 460 genes did not reveal enriched groups of genes, or ones belonging to the same metabolic pathways. However, of these 460 genes, 18 and 36 are encoded by the mitochondrial and chloroplast genomes, respectively, and thus were not represented on the Affymetrix ATH1 array.

**Figure 2:**
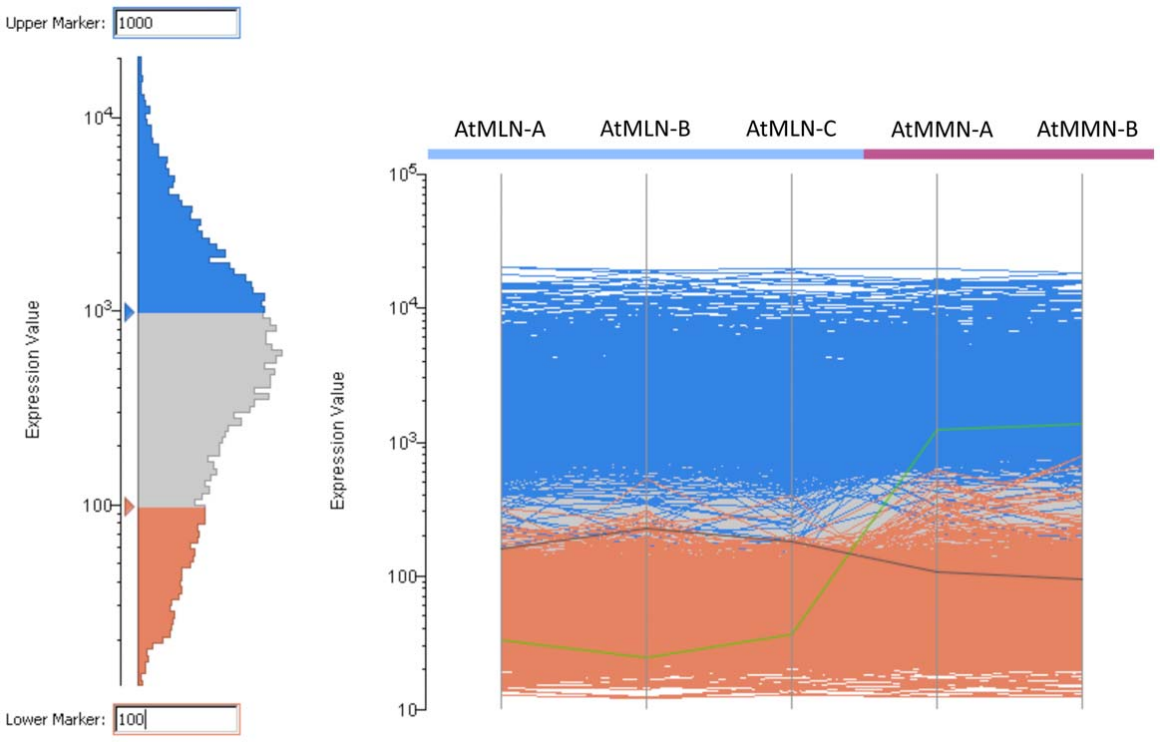
Expression level of Arabidopsis orthologs represented by *B. rapa* EST hits. The 3,678 genes represented by *B. rapa* ESTs, and confidently expressed in Arabidopsis nectaries were examined for their expression levels. Of these genes, 798 had intensity values under 100, 1,477 between 100 and 1,000, and 1,401 were above 1,000. Biological replicates described in [17] are indicated by AtMLN-A, AtMLN-B, AtMLN-C, AtMMN-A, and AtMMN-B.

Regarding expression levels in Arabidopsis nectaries, of the 3,678 presumptive Arabidopsis orthologs represented by *B. rapa* ESTs, 798 had intensity values under 100, 1,477 between 100 and 1,000, and 1,401 were above 1,000 (graphically represented in Fig. 2). The microarray probe set signal intensities had been normalized to a median of 100 [17]; therefore, more than 60% of the 3,678 Arabidopsis nectary-expressed orthologs had signal intensities greater than the median. Thus, it appears that the *B. rapa* nectary cDNA library construction methods likely had a preference for highly expressed genes, even though this effort also captured a significant number of low expressed genes. Similar expression profiles were also observed for each individual library (both normalized and non-normalized), not just the unigene set (data not shown).

Finally, it should also be noted that the *Brassica* sp. genome underwent triplication prior to divergence and then becoming the current diploid species [22], [23]. Thus it is likely that a number of Arabidopsis othologues may be represented by multiple *B. rapa* paralogs found within our EST sequences. We attempted to find potentially paralogous sequences within our EST data; however, we were unable to confidently identify examples.

### Digital Expression Profiling in Median versus Lateral Nectaries

As mentioned above, random sequencing of cDNA libraries permits comparison of gene expression between two tissues (or treatments) through the evaluation of the number of redundant or overlapping sequences identified [19]. If ESTs representing a given gene are identified a large number of times in one cDNA library but not another, it can be deduced that the gene represented by those tags is highly expressed in one tissue (or treatment) and not the other. For this study, non-normalized libraries were created for both median (MMN-1) and lateral nectaries (MLN-1). This is significant, as even though median and lateral nectaries appear to share similar developmental and morphological features, lateral nectaries secrete >95% of total carbohydrate in most Brassicaceae flowers [8], with median nectaries often being largely non-functional [11]. We hypothesize that differential gene expression between median and lateral nectaries is at least partially responsible for the observed disparity in nectar production between these two sets of organs.

**Figure 3:**
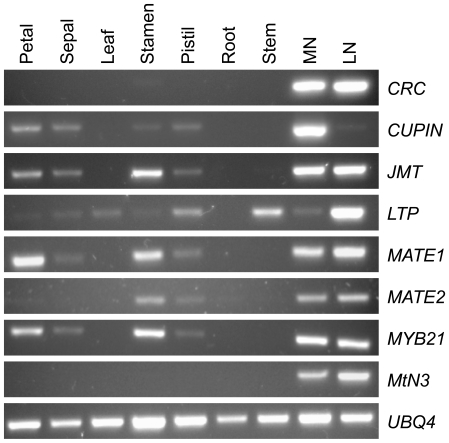
RT PCR of putative *B. rapa* orthologs to Arabidopsis nectary-enriched genes and confirmation of differential gene expression between median and lateral nectaries. Microarrays were previously used to identify Arabidopsis nectary-enriched genes [17]. Here, RT PCR was used to verify *B. rapa* orthologs for eight of these genes, as well as differential expression between median and lateral nectaries, as identified by EST hit analysis. The results for the following *B. rapa* contigs listed in Table S7 are displayed (Arabidopsis orthologs are listed in parentheses): All.Contig_261 (*CRC*, At1g69180), All.Contig_175 (cupin family protein, At1g74820), All.Contig_244 (*JMT*, At1g19640), All.Contig_14 (lipid transfer protein, LTP; At5g55450), All.Contig_1907 (MATE efflux protein, At1g23300), All.Contig_1730 (MATE efflux protein, At1g51340), All.Contig_581 (*MYB21*, At5g40360), All.Contig_267 (*MtN3*, At2g39060). An ortholog to Arabidopsis UBIQUITIN 4 (All.Contig_1549; UBQ4, At4g05320) was used as a constitutively expressed control. All images shown are the results derived from 27 to 30 cycles of PCR. Expression patterns were also previously confirmed for orthologs to At2g36190 (All.Contig_537; [16]) and At1g77110 (All.Contig_963; Ruhlmann and Carter, *in preparation*). It should be noted that it is possible that the observed bands may represent the expression of multiple paralogous genes.

To identify genes potentially differentially expressed between lateral and median nectaries, we initially compared the ratios of ESTs represented within contig and singleton sequences derived from the non-normalized MLN-1 and MMN-1 cDNA libraries that generated hits against common Arabidopsis genes (as identified by BLAST analysis). Full data for the number of ESTs within contigs and singletons that generated hits against distinct Arabidopsis genes for each library (both normalized and non-normalized) are displayed in Table S8. We noticed that the ratios of gene expression between median and lateral nectary ESTs were often conserved between the normalized and non-normalized libraries. As such, ten genes displaying some of the largest differences in EST hit numbers between median and lateral nectaries for both sets of libraries are listed in Table 2. Reverse transcription polymerase chain reaction (RT PCR) was later used to verify differential expression for three of these genes (see below, Fig. 3), with a fourth being previously demonstrated (At1g77110; Ruhlmann and Carter, *in preparation*). As an alternative to the analyses described above, blastx searches for each trimmed EST sequence, without prior contig assembly, were also performed against all Arabidopsis proteins, which generated similar EST hit ratios for the same genes as the analyses above. Full BLAST results and summarized hit numbers for this alternative analysis are available in Table S9.

**Table 2:** Some putative orthologs to Arabidopsis genes displaying differential expression between nectary type by EST hit number.

		No. of ESTs generating hits against Arabidopsis locus^b^							
Arabidopsis locus^a^	TAIR annotation	MLN-1	MMN-1	MLN-2	MMN-2	All MLN	All MMN	AtMLM:: AtMMN^c^	P-value
At2g39060	nodulin MtN3 family protein^d^	78	11	12	15	90	26	1.08	0.50
At5g55450	protease inhibitor/seed storage/lipid transfer protein (LTP) family protein^d^	19	0	8	0	27	0	0.89	0.61
At3g52930	fructose-bisphosphate aldolase, putative	10	2	8	3	18	5	0.80	0.22
At1g60950	FED A (FERREDOXIN 2)	10	1	6	1	16	2	0.82	0.18
At4g28240	Wound-responsive protein-related	7	1	3	0	10	1	0.79	0.53
At1g74820	cupin family protein^d^	1	244	4	75	5	319	0.02	0.00
At2g42530	COR15B	0	20	0	4	0	24	0.88	0.16
At1g77110	PIN6 (PIN-FORMED 6)	0	2	0	7	0	9	0.93	0.62
At5g02120	OHP (ONE HELIX PROTEIN)	1	13	1	3	2	16	1.44	0.28
At5g15800	SEP1 (SEPALLATA1)	1	7	0	3	1	10	0.71	0.05

a Locus identified by blastx searches of *B. rapa* contigs against Arabidopsis proteins (TAIR9 annotation release).

b Number of ESTs within contigs and singleton sequences generating hits against Arabidopsis loci, listed by individual library.

c Affymetrix ATH1 mean signal probe ratio; original raw data from [17].

d Differential expression verified by RT PCR, results shown in Fig. 3.

e Expression pattern also previously verified for ortholog to A1g77110 (Ruhlmann and Carter, *in preparation*).

It should be noted that all genes displaying differences in EST hit numbers between median and lateral nectaries may not represent true differential expression. A more confident analysis would require much more extensive sequencing data, or the use of microarrays. At a minimum, readers are advised to use RT PCR to validate differential expression based upon EST hit number prior to conducting downstream experimentation. To partially address this issue, and to examine if differential expression patterns may be conserved between the median and lateral nectaries of *B. rapa* and Arabidopsis, we compared *B. rapa* EST hit numbers to our previous Arabidopsis nectary microarray data [17]. The mean probe set signal intensity for both Arabidopsis median and lateral nectaries, and their signal ratios (measure of differential expression) is presented with the EST hit numbers in Table S8, along with select genes in Table 2.

Surprisingly, there was very little conservation of differential gene expression between median and lateral nectaries as identified by both *B. rapa* EST hit number and Arabidopsis microarray (see Table 2, Table S8). For example, 141 presumptive *B. rapa* genes were identified as having two-fold or greater total EST hits in one nectary type than the other (with a minimum of 5 ESTs present in at least one nectary type; see second and third tabs of Table S8). However, only six of these 141 genes also displayed statistically significant differences in expression between Arabidopsis MMN and MLN via microarray analysis (all were higher in MMN than MLN; see second tab of Table S8). Despite these results, we found that several genes displaying differential expression by EST hit number (orthologs to At2g39060, At5g55450, and At1g77110) were verified to be upregulated in one type of *B. rapa* nectary over the other via RT PCR (Fig. 3), even though these differences were not observed in Arabidopsis via microarray (Table 2). Thus at least some of the observed differences in expression between the lateral and median nectaries of *B. rapa* and Arabidopsis do represent true biological variation. Interestingly, it was previously reported that microarray and EST analyses from the same RNA samples can give varying results for which genes are differentially expressed [24]. Therefore, there is perhaps some precedence for our observation that differential expression between median and lateral nectaries *appears* to not be particularly conserved between Arabidopsis and *B. rapa*, which may be due in large part to platform differences (i.e., EST *versus* microarray analysis).

### Identification of Orthologs to Known Arabidopsis Nectary-Enriched Genes

We previously performed an analysis of the Arabidopsis nectary transcriptome via microarray, which allowed the identification of a large number of genes preferentially expressed in nectaries [17]. Thus, in addition to the digital expression profiling described above, we were able to identify putative *B. rapa* orthologs to 207 known Arabidopsis nectary-enriched genes via BLAST searches [three-fold, see Tables S10 (MLN) & S11 (MMN)] with ten of the most nectary-specific Arabidopsis genes, and corresponding *B. rapa* EST hit numbers, being listed in Table 3.

**Table 3:** *B. rapa* EST analysis identified putative orthologs to highly nectary-enriched Arabidopsis genes.

Arabidopsis locus	TAIR Annotation	AtMLN/Ref^a^	AtMMN/Ref^a^	No. of ESTs^b^
At1g74820	cupin family protein^c^	6.20	405.88	324
At1g65980	peroxiredoxin type 2, putative	4.34	0.28	220
At2g39060	nodulin MtN3 family protein^c^	193.32	179.31	114
At2g36190	beta-fructosidase, putative^d^	66.63	62.00	59
At1g23010	multi-copper oxidase type I family protein	26.07	26.49	27
At3g27810	myb family transcription factor^c^	10.59	9.50	23
At1g69180	transcription factor CRC (CRABS CLAW)	198.09	251.69	22
At1g19640	S-adenosyl-L-methionine:jasmonic acid carboxyl methyltransferase (JMT)^c^	33.04	7.24	15
At2g42830	agamous-like MADS box protein AGL5	42.37	37.65	14
At4g12530	protease inhibitor/seed storage/lipid transfer protein (LTP) family protein	376.33	352.76	14
At1g77110	PIN6 (PIN-FORMED 6); auxin:hydrogen symporter/transporter^d^	84.81	90.87	8
At1g51340	MATE efflux family protein^c^	37.65	32.61	1
At1g23300	MATE efflux family protein^c^	171.12	58.88	1

a Fold difference in normalized microarray probe signal intensity for Arabidopsis MLN and MMN over the average of seventeen individual reference tissues (described in [17]). All genes listed here displayed a minimum two-fold increase in mean probe set signal intensity in MLN and/or MMN over each individual reference tissue, along with t-test p-values and false discovery rate (FDR) q-values of <0.05. Full lists of genes meeting these cutoff criteria via Arabidopsis microarray, along with average probe intensities and p and q-values for individual nectary samples, and corresponding number of *B. rapa* EST hits, are available in Tables S10 (MLN) and S11 (MMN). A total of 207 putative *B. rapa* orthologs to Arabidopsis nectary-enriched genes (MLN and/or MMN) were identified through this analysis.

b Number of *B. rapa* ESTs from Table S9 (ESTs from all libraries combined) generating hits against Arabidopsis locus.

c Nectary-enriched expression of ortholog in *B. rapa* verified by RT PCR, results shown in Fig. 3.

d Expression patterns also previously confirmed for orthologs to At2g36190 [16] and At1g77110 (Ruhlmann and Carter, *in preparation*).

To determine if the presumptive *B. rapa* orthologs had similar expression profiles to their Arabidopsis counterparts, we performed reverse transcription polymerase chain reaction (RT PCR). Results shown in Fig. 3 confirmed the nectary-enriched expression for genes represented by eight contig sequences [with two others previously demonstrated: orthologs to At2g36190 [16] and At1g77110 (Ruhlmann and Carter, *in preparation*)], and suggest they are true orthologs to the Arabidopsis nectary-expressed genes. While sequence analysis was unable to identify potentially paralogous sequences, it is important to note that the bands observed in Fig. 3 could represent the expression patterns of multiple related genes. As mentioned previously, these results also confirmed differential expression of three genes between median and lateral nectaries [orthologs to At1g74820 (cupin family protein), At4g12530 (lipid transfer protein, LTP) and At2g39060 (MtN3)], as initially identified by EST hit number, with a fourth (At1g77110) also being previously confirmed (Ruhlmann and Carter, *in preparation*). Since Arabidopsis and *B. rapa* are closely related, and appear to share genes with nectary-enriched expression profiles, it is likely that these two species share similar mechanisms of nectar production.

### *B. rapa* Nectaries Are Enriched for ESTs Representing Genes Involved in Photosynthesis

To potentially glean more biological information from the sequence data, we examined the GO biological process annotations for ESTs represented within *B. rapa* nectaries, along with 8,771 *B. rapa* whole flower ESTs, 8,265 *B. rapa* root ESTs, and 12,448 tobacco nectary ESTs currently available in dbEST. The presumptive Arabidopsis orthologs to all of these sequences were independently identified via blastx searches, and GO Biological Process categories were then extracted from the newest Affymetrix annotation file (ATH1-121501 Annotations; 3/12/09). Fisher's Exact Test in Expressionist software (GeneData) was used to determine the significance of Arabidopsis orthologous genes of the ESTs from different tissues of *B. rapa*, and tobacco nectaries, seemingly overrepresented in a particular GO category when compared against all genes contained in said GO category. In each case Fisher's test indicated whether it was possible to reject the null hypothesis that the observed differences are due to chance.

Results from this analysis suggested that *B. rapa* MLN ESTs for photosynthesis processes (see image A in Table S12) are extremely significantly enriched (p-value 10^−24^). Even though photosynthesis processes are also the most enriched processes of MMN, it is of lower significance (10^−14^) when compared to MLN (see image B in Table S12). The photosynthesis processes are also enriched in *B. rapa* whole flowers, 10^−19^, (see image C in Table S12) and tobacco nectaries, 10^−14^, (see image D in Table S12), but other processes are more dominant than photosynthesis processes in these tissues. Not surprisingly, photosynthesis processes are not enriched in *B. rapa* roots (see image E in Table S12). The *B. rapa* nectary-expressed genes putatively involved in photosynthesis (n = 55), along with graphical representations of all processes apparently enriched or depleted represented within the ESTs of each of the tissues examined, can be found in Table S12. Interestingly, every one of the 55 Arabidopsis photosynthesis loci represented by *B. rapa* ESTs were also confidently expressed in Arabidopsis nectaries via microarray, with more than 90% of these genes displaying probe set signal values >1,000 (median intensity was scaled to 100). Therefore, these photosynthesis process genes appear to be highly expressed in both *B. rapa* and Arabidopsis nectaries.

The results described above are noteworthy because the source of nectar carbohydrate in *Brassica* flowers is still somewhat in question. It is generally thought that most nectar carbohydrate for the majority of nectar-secreting plants is first produced in source tissues (e.g., leaves) and then transported via phloem to the nectaries (sink tissues) [5], [25], [26]; however, it has been suggested that some nectar carbohydrate may be produced *in situ* by *Brassica* nectaries via direct photosynthesis [12]. Thus, genes involved in photosynthetic processes, represented by nectary ESTs identified here, may be directly responsible for the generation of sugars constituting *B. rapa* nectar, and ultimately contributing to pollinator visitation and reproductive success.

### Conclusions

From this study, sequences representing a minimum of 4,100 unique genes (possibly many more due to paralog issues) expressed in both the median and lateral nectaries of *B. rapa* were putatively identified by EST analysis. Comparisons of the number of ESTs representing unique transcripts also allowed the discovery of several genes differentially expressed between median and lateral nectaries, which were not previously observed in Arabidopsis via microarray analysis. Moreover, a large number of putative orthologs to Arabidopsis nectary-enriched genes were identified by BLAST searches, with eight of these orthologs being verified by RT PCR analysis. Since Arabidopsis flowers are very small and produce very little nectar, the work described here provides clones and sequence information that may be useful for discovering genes involved in nectar production in *B. rapa*, a model system that produces relatively large volumes of floral nectar. Since *B. rapa* is highly dependent on pollinators to achieve efficient pollination, this work may also allow the identification of genes with impacts on overall reproductive fitness via their roles in mediating nectary form and function.

## Materials and Methods

### Plant Materials and Growth

Rapid-cycling *Brassica rapa* (CrGC 1-33) was used in this study. Plants were grown in individual pots on a peat-based growth medium with vermiculite and perlite (Pro-Mix BX; Premier Horticulture, Rivière-du-Loup, Quebec, Canada). All plant growth was performed with a 16 hr light/8 hr dark cycle, photosynthetic photon flux of 150 µmol m^−2^ s^−1^, and temperature of 21°C.

### cDNA Library Construction

Median and lateral nectaries were manually dissected from open *B. rapa* flowers (equivalent of Stage 14–15 in Arabidopsis [27]) and pooled in separate tubes containing RNAlater™ solution (Ambion, Austin, TX) on ice, and stored at −20°C prior to RNA extraction. Individual samples containing ca. 250 nectaries were processed for RNA isolation with Stratagene's Absolutely RNA Miniprep Kit (#400800). Total RNA quality was assessed by standard UV spectrophotometry and agarose gel electrophoresis; individual preparations yielded ∼10 micrograms of total RNA. Two non-normalized cDNA libraries (MMN-1 and MLN-1) were generated from 1 microgram of total RNA with the Creator™ SMART™ cDNA Library Construction Kit (Clontech #634903) according to the manufacturers directions (LD PCR method). A second set of normalized libraries (MMN-2 and MLN-2) were prepared by Creative Genomics, Corp. (Port Jefferson Station, New York) from 15 micrograms of total RNA by first amplifying cDNA with the Creator™ SMART™ cDNA Library Construction Kit, and then normalizing the product with the Trimmer-direct Kit (Evrogen #NK002). All cDNA fragments for both sets of libraries were ligated into the *Sfi*I A and B sites of pDNR-LIB, and transformed into either DH5α (MLN-1 and MMN-1 libraries) or DH10B (MLN-2 and MMN-2 libraries). The resultant clones from each library were examined for quality (average insert size and percent of clones without inserts) via plasmid DNA isolation, *Sfi*I digestion, and 1% agarose gel electrophoresis.

### DNA Sequencing

*E. coli* carrying clones from each of the cDNA libraries were robotically processed, including: plating, colony picking, growth in 96 well format, plasmid isolation, and random sequencing from the 5′ end via dideoxy sequencing. These steps were performed at either the University of Washington High Throughput Genomics Unit (MLN-1 and MMN-1 libraries) or at Creative Genomics, Corp. (MLN-2 and MMN-2 libraries). All sequencing was performed with the universal M13 Reverse primer (5′-CAGGAAACAGCTATGACC-3′).

### Sequence Processing and Analysis

All sequencing reads were trimmed of poor quality regions, and contaminating vector and linker sequences, and then assembled into contigs with Lasergene SeqMan Pro© version 8.0.2 software (DNASTAR, Inc.) with ProAssembler default settings (match size = 25, minimum match percentage = 80, match spacing 150, minimum sequence length = 100, gap penalty = 0, gap length penalty = 0.70, and maximum mismatch end bases = 15). To identify potential functions of the resultant contigs and singletons, a local version of the Basic Local Alignment Search Tool (BLAST) was used (v. 2.2.20). EST and contig sequences were subjected to translated (blastx) searches against Arabidopsis proteins (TAIR9 annotation release [21]) with default settings, including: use of the BLOSUM62 matrix; expect threshold of 10; and, gap costs of existence: 11 and extension: 1. Each independent trimmed EST was deposited into the National Center for Biotechnology Information's (NCBI) GenBank and dbEST databases, with the resultant accession numbers for each library being presented in Tables S1, S2, S3, and S4.

### RT PCR Analyses

The RNAqueous-Micro® micro scale RNA isolation kit (Ambion, Austin, TX) was used, in conjunction with Plant RNA Isolation Aid (Ambion, Austin, TX), to extract RNA from *B. rapa* tissues. For floral tissues, RNA was extracted from the equivalent of Stage 14–15 Arabidopsis flowers [27]. Standard agarose gel electrophoresis and UV spectrophotometry were used to evaluate RNA quality for all samples. Reverse transcription polymerase chain reaction (RT PCR) was used to examine the presence of transcripts with Promega Corporation's (Madison, WI, USA) Reverse Transcription System (A3500), in conjunction with GoTaq Green Master Mix (Promega, M7122), according to the manufacturer's instructions. All primers used for RT PCR analyses are listed in Table S13.

## Supporting Information

Table S1 MLN-1 EST and contig sequences with blastx results. The first tab contains MLN-1 contig sequences and blastx results for each contig. The second tab contains all MLN-1 trimmed ESTs, GenBank and dbEST accession numbers, and also indicates which ESTs formed which contigs.
(3.18 MB XLS)

Table S2 MMN-1 EST and contig sequences with blastx results. The first tab contains MMN-1 contig sequences and blastx results for each contig. The second tab contains all MMN-1 trimmed ESTs, GenBank and dbEST accession numbers, and also indicates which ESTs formed which contigs.
(3.24 MB XLS)

Table S3 MLN-2 EST and contig sequences with blastx results. The first tab contains MLN-2 contig sequences and blastx results for each contig. The second tab contains all MLN-2 trimmed ESTs, GenBank and dbEST accession numbers, and also indicates which ESTs formed which contigs.
(4.97 MB XLS)

Table S4 MMN-2 EST and contig sequences with blastx results. The first tab contains MMN-2 contig sequences and blastx results for each contig. The second tab contains all MMN-2 trimmed ESTs, GenBank and dbEST accession numbers, and also indicates which ESTs formed which contigs.
(3.39 MB XLS)

Table S5 Contig sequences for all MLN ESTs combined (MLN-1 + MLN-2) and blastx results for each contig. The second tab contains all MLN trimmed ESTs, and also indicates which ESTs formed which contigs.
(5.69 MB XLS)

Table S6 Contig sequences of all MMN ESTs combined (MMN-1 + MMN-2) and blastx results for each contig. The second tab contains all MMN trimmed ESTs, and also indicates which ESTs formed which contigs.
(6.24 MB XLS)

Table S7 Contig sequences generated for all ESTs combined (unigene set; MLN-1 + MLN-2 + MMN-1 + MMN-2) and blastx results for each contig. The second tab indicates which ESTs formed which contigs.
(7.68 MB XLS)

Table S8 Summarized blastx hits for contigs and singletons, with Arabidopsis ortholog microarray expression data. First tab: number of B. rapa ESTs within contigs generating hits against Arabidopsis loci for each cDNA library. Loci highlighted in yellow (n = 460) were not found to be significantly expressed in Arabidopsis nectaries previously (Kram et al., BMC Plant Biol, 2009). Eighteen mitochondrial (green) and 36 plastidial (gray) encoded orthologs not represented in the ATH1 array are also highlighted. All non-highlighted genes (n = 3,678, or 89% of the 4,138 unigenes) were confidently expressed in Arabidopsis nectaries. Column M contains the expression ratios of AtMLN vs AtMMN derived from Arabidopsis microarray experiments, with column N showing the t-test significance between AtMLN vs AtMMN expression values (data from Kram et al., BMC Plant Biol, 2009). Second tab: Genes upregulated in MMN by EST hit number. All genes showing a 2-fold or greater number of EST hits in MMN (minimum of 5 ESTs required) than MLN are displayed. Genes highlighted in pink displayed 1.5 fold or greater expression in Arabidopsis MMN over MLN, with t-test p-values <0.1, as determined by microarray. Third tab: Genes upregulated in MLN by EST hit number. All genes showing a 2-fold or greater number of EST hits in MLN (minimum of 5 ESTs required) over MMN are displayed. None of these genes displayed 1.5 fold or greater expression in Arabidopsis MLN over MMN, with a t-test p-value <0.1, as determined by microarray.
(2.56 MB XLS)

Table S9 Number of ESTs generating hits against same Arabidopsis loci for each cDNA library. Contains raw blastx results.
(4.64 MB XLS)

Table S10 Identification of B. rapa orthologis to Arabidopsis nectary-enriched genes. All genes listed here displayed a two-fold increase in mean probe set signal intensity in Arabidopsis MLN over each individual reference tissue, along with t-test p-values and false discovery rate (FDR) q-values of <0.05. Each of these Arabidopsis genes had orthologs represented by B. rapa ESTs identified via BLAST searches. The corresponding number of B. rapa EST hits, from Table S9, are also presented.
(0.34 MB XLS)

Table S11 Identification of B. rapa orthologs to Arabidopsis nectary-enriched genes. All genes listed here displayed a two-fold increase in mean probe set signal intensity in Arabidopsis MMN over each individual reference tissue, along with t-test p-values and false discovery rate (FDR) q-values of <0.05. Each of these Arabidopsis genes had orthologs represented by B. rapa ESTs identified via BLAST searches. The corresponding number of B. rapa EST hits, from Table S9, are also presented.
(0.33 MB XLS)

Table S12 Arabidopsis orthologs to B. rapa MLN-expressed genes putatively involved in photosynthesis,as identified by GO analysis. Also presented are images of all GO processes apparently enriched or depleted within B. rapa mature lateral nectaries, mature median nectaries, whole flowers, roots, and tobacco nectaries.
(5.18 MB XLS)

Table S13 Oligonucleotides used for RT PCR analyses
(0.02 MB XLS)

Video S1 Example of B. rapa nectary excision
(1.29 MB MOV)
